# Microstructural Response in Friction Stir Additive Manufacturing of 5A06 Aluminum Alloy

**DOI:** 10.3390/ma18081713

**Published:** 2025-04-09

**Authors:** Yaobang Zhao, Bo Chen, Wukai Li, Junchen Li, Junmiao Shi, Baiming Wang, Feng Jin

**Affiliations:** 1 Shanghai Shenjian Precision Machinery Technology Co., Ltd., Shanghai 201600, China; zyb0916@163.com (Y.Z.); bobomailk@163.com (B.C.); luk_2014@163.com (W.L.); lijunchen123@126.com (J.L.); 2 Key Laboratory of Pressure Systems and Safety, Ministry of Education, East China University of Science and Technology, Shanghai 200237, China; shijunmiao@ecust.edu.cn; 3 School of Energy and Power Engineering, Jiangsu University, Zhenjiang 212013, China; 3220212310@stmail.ujs.edu.cn; 4 School of Materials Science and Engineering, Jiangsu University, Zhenjiang 212013, China

**Keywords:** friction stir additive manufacturing, microstructural response, AA5A06, carbon nanotubes, EBSD

## Abstract

Friction stir additive manufacturing (FSAM) technology is the ideal technique for aluminum alloy additive manufacturing from the perspective of defect control, microstructure regulation, and performance optimization. However, there is limited systematic fundamental research on the aluminum alloy FSAM. This study implemented a consumable-tool-based 5A06 FSAM process. By incorporating carbon nanotubes during the FSAM process, our research investigated its impact on grain refinement and the performance of the additive structure. The results show that the well-formed additive structure is composed of multiple layers of stirred metal. The microstructure of the additive structure of AA5A06 consists of refined recrystallized grains and deformed grains within each layer, while the interface between layers is composed of a finer-grain band, with an average grain size of 6 µm, whose tensile strength ranges from 225 MPa to 260 MPa, with an elongation of 26% to 32%. After the addition of carbon nanotubes, although the grain size was refined to 2 µm, there was no improvement in tensile strength, and the elongation was reduced. The tensile strength now ranges from 225 MPa to 270 MPa, with elongation between 12% and 16%.

## 1. Introduction

Additive manufacturing (AM), also known as three-dimensional (3D) printing, offers several advantages across a range of industries, from the aerospace and automotive industries to those responsible for healthcare and consumer products [1,2]. AM offers many advantages such as design freedom, material efficiency, faster time-to-market, reduced waste, opportunities to recycle materials, and the ability to produce customized parts [3,4,5,6]. These benefits make AM a powerful tool for industries seeking to innovate, optimize production, and improve sustainability. 5A06 is a wrought aluminum alloy known for its good strength-to-weight ratio, which is essential in the aerospace and automotive industries, as well as in marine applications. It has excellent toughness and is resistant to corrosion, making it suitable for both structural and functional components [7,8]. In aerospace, the ability to print lightweight, high-strength, and complex geometries with 5A06 aluminum alloy can significantly reduce the weight of aircraft components, which is a critical factor in improving fuel efficiency and performance [9,10]. AM can enable the production of parts with optimized designs that traditional manufacturing methods cannot achieve.

Additive manufacturing technologies, which originated in the 1980s, have undergone significant development over recent decades. Processes like wire arc additive manufacturing (WAAM), laser powder bed fusion (L-PBF), and binder jetting have revolutionized the manufacturing of aluminum alloys. This evolution has now advanced toward solid-state techniques, including friction stir additive manufacturing (FSAM). WAAM was developed around the 1990s. Laser powder bed fusion (L-PBF) was developed by Fraunhofer around 1995 [11] and laser powder directed energy deposition (LP-DED) was licensed by Sandia National Laboratory around 1997 [12]. Thereafter, electron-beam powder bed fusion (EB-PBF) was commercialized by Arcam in Sweden around 2001 [13]. Finally, additive friction stir deposition (AFSD) was patented by MELDTM Manufacturing Corporation [14]. As one may have noticed, the AM process of aluminum alloys has developed from an AM process with solidification characteristics to a solid-state AM process. Accounting for this, solidification defects such as pores and cracks are the problems that most urgently need to be resolved for AM methods based on the melting–solidification process; these are the issues that have been focused on in previous studies. Based on in situ X-ray imaging [15], six pore formation mechanisms have been identified: pore formation due to raw material, pore induction by small holes, pore formation caused by volatile substances or the expansion of trapped gas, pore capture by surface fluctuations, pore formation due to fluctuations in recessed areas, and pore formation caused by cracks. Currently, pores can only be controlled through online monitoring methods. Due to the solidification process, aluminum alloy AM often suffers from cracks. The formation of cracks is typically related to solidification shrinkage and a relatively large solidification range [15]. Cracks can be classified as hot cracks and cold cracks [16]. Hot cracks are believed to be caused by shrinkage during the solidification process. In the final stage of solidification, unstable and supercooled solute-rich melt generates dendritic inter-dendritic channels with thin liquid layers. Hot cracks are usually located along grain boundaries, while cold cracks originate from the extension of hot cracks along vertical grain boundaries. To eliminate cracks, considerable efforts have been made, including adjustments to printing parameters [17], printing conditions (such as build plate preheating) [18], compositional modifications [19], and inoculation treatments. Notably, grain refinement induced by inoculation treatment has become a more popular method of crack repair [20,21]. The basic design concept of inoculation treatment is to introduce secondary particles Al3X (X = Zr, Sc, Ti, and Nb) that are congruent with the Al matrix to refine the grains, thereby preventing crack formation and propagation and enhancing grain boundary strengthening. Compared to coarse columnar grains, fine equiaxed grains are more effective in eliminating cracks. In summary, the AM process of aluminum alloys is evolving from a solidification-based approach to a solid-state AM process. FSAM technology has emerged as a solution to some of the limitations of conventional fusion-based additive manufacturing methods. Traditional methods like laser and electron beam-based technologies rely on melting the material to fuse it layer by layer, which can lead to issues like thermal distortion, residual stresses, and poor material properties. FSAM, by contrast, works by deforming the material through solid-state processes, allowing for a more controlled buildup of layers without the need for high temperatures that would typically cause melting.

FSAM is based on the solid-state joining process of friction stir welding (FSW). During FSW, a rotating tool with a specially designed probe and shoulder is used to generate frictional heat and mechanical stirring in the workpiece material. This allows for the plastic deformation and mixing of the material at a temperature below its melting point, resulting in a strong and defect-free bond [22,23,24,25]. In FSAM, this process is adapted for AM. Commonly, material is deposited layer by layer, and the same tool rotates and moves along the deposition path. The tool moves through previously deposited material, mechanically stirring it to consolidate the new material with the underlying layer, forming a strong, homogeneous bond. FSAM is a new technique for aluminum alloy additive manufacturing, and current basic research on this technique is quite limited. Chen proposed a wire-based FSAM process, which can realize the solid-state manufacturing of large metallic structures via continuous feeding of wire materials [26]. Similarly, AM can also be achieved by consuming stirring tools. Compared with the traditional laser- or electron beam-based AM technologies, FSAM shows many advantages. For example, FSAM technologies generally do not require such stringent environmental controls, i.e., high vacuum or controlled atmospheres of laser- or electron beam-based AM technologies, making them more cost-effective and adaptable to a broader range of industrial environments. FSAM can achieve faster deposition rates, allowing for larger components to be produced more efficiently. This is especially useful for industries like the aerospace or automotive industries, where large, complex parts are often required [27]. Most importantly, FSAM can control the performance of additive structures by refining the grain size. However, there is limited systematic fundamental research on the aluminum alloy FSAM. For example, the limit of grain refinement achievable in aluminum alloy FSAM and its impact on the performance of the additive structure are in need of further study.

In this study, FSAM of 5A06 aluminum alloy was achieved. Carbon nanotubes were then added during the FSAM process to refine the grain structure, and the effect of carbon nanotubes addition on grain refinement was investigated. Next, Electron Backscatter Diffraction (EBSD) was employed to characterize the microstructures of both carbon nanotubes-free and carbon nanotubes-containing additive structures, and their effects on the tensile properties were analyzed. This study will provide fundamental data for FSAM and lay the foundation for the application of the FSAM process.

## 2. Experimental Procedures

**Figure 1:**
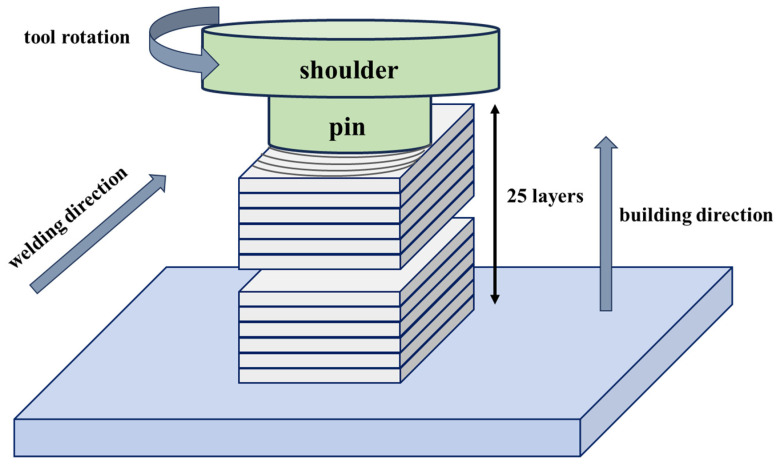
Schematic diagram of the friction stir additive manufacturing process.

The FSAM process in this study uses the consumption of the stirring tool to achieve additive deposition, as shown in Figure 1. The substrate is selected as a forged AA5A06 plate. First, a 5A06 aluminum alloy rod is chosen as the stirring tool to achieve the additive process with 25 layers of stirring on the 5A06 plate. A 5A06 aluminum alloy stirring tool containing 1.5 wt.% carbon nanotubes was prefabricated using solid-state powder metallurgy. Then, the tool with carbon nanotubes was used to manufacture the additive deposition of a 5A06 bulk material with carbon nanotubes on the AA5A06 plate by friction stir processing. The process parameters used during the FSAM were a spindle speed of 300 rpm, tool travel speed of 100 mm/min, and axial pressure of 2 MPa.

After obtaining the samples of FSAM, slice sampling was conducted on the additive structure. First, the morphology of the additive structure was observed. Subsequently, considering the possible microstructure inhomogeneity within the additive structure, EBSD was employed to examine and characterize the upper, middle, and lower regions of the additive structure, as well as the interface between the additive structure and the substrate. The samples prepared for EBSD observation were mechanically polished first, followed by the application of ion beam surface stress relief techniques to bombard the surface of the samples and remove surface stress. EBSD analysis was conducted using the OXFORD NORDLYS X-MAX system and HKL-Channel5 software. The step size for the EBSD scan was set to 0.1 µm, operating at 20 kV with an inclination angle of 70 degrees. Inverse pole figure (IPF) maps were utilized to characterize the microstructures, with boundaries between grains that had different orientations of 15° or higher defined as high-angle boundaries. The misorientation distribution histogram was used to quantify the misorientation information in the EBSD region, which helped us to analyze the microstructural behavior of friction stir additive manufacturing (i.e., recrystallization). Subsequently, the grains in the EBSD region were statistically analyzed to characterize the grain size information of the FSAM.

**Figure 2:**
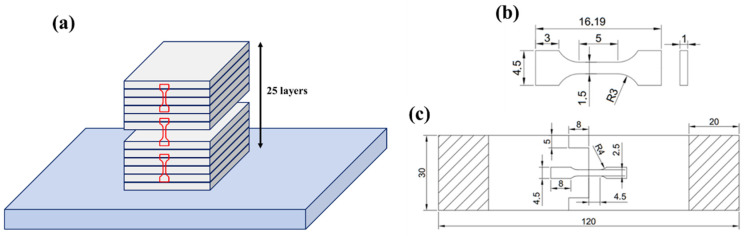
Schematic diagram of sampling location (**a**) and the detailed dimensions of tested samples (**b**) and the self-designed fixture (**c**).

Thereafter, considering the potential performance inhomogeneity across the upper, middle, and lower regions (i.e., as schematically shown in Figure 2a) of the friction stir additive structure, tensile samples were taken from these regions. The details of the sample dimensions are shown in Figure 2b. The sample surfaces were then mechanically polished to eliminate the effects of surface damage on tensile performance. Finally, tensile tests were conducted using a self-designed fixture, as shown in Figure 2c. The tensile test was conducted on a tensile test machine (INSTRON 3382) with a cross-head speed of 0.5 mm/min.

## 3. Results and Discussion

### 3.1. Morphology of Friction Stir Additive Manufactured AA5A06

**Figure 3:**
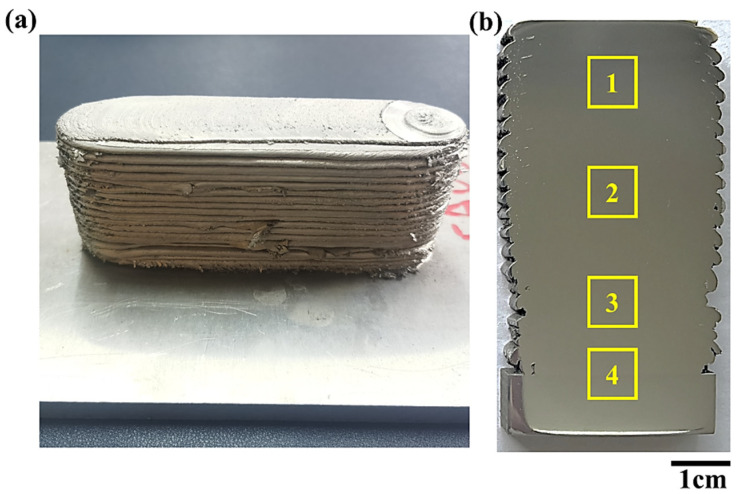
Morphology of friction stir additive manufactured AA5A06: (**a**) appearance, and (**b**) cross-sectional morphology.

Figure 3 shows the macro morphology of friction stir additive manufactured AA5A06. Generally, from the appearance shown in Figure 3a, the additive manufactured sample is composed of layers of friction-stirred aluminum alloy. Figure 3b displays the morphology of the cross-section, which intuitively demonstrates the effect of the layer-by-layer (~25 layers) metal stacking. As shown in Figure 3b, the quality of the additive manufacturing is good, with no noticeable macro defects such as porosity. There are only some minor defects at the junctions between the layers (at the edges), which may be caused by the metal flow generated at the outer edge of the tool during the friction stir process.

### 3.2. Microstructural Responses of Friction Stir Additive Manufactured AA5A06

**Figure 4:**
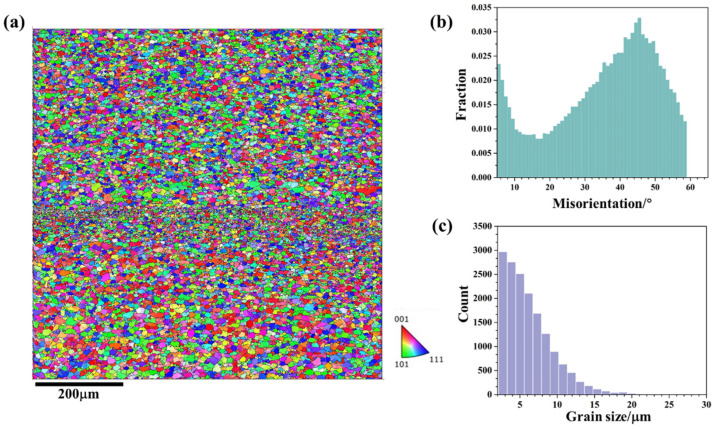
Microstructural response at upper region (zone 1 in Figure 3) of friction stir additive manufactured AA5A06: (**a**) IPF map, (**b**) misorientation distribution map, and (**c**) grain size distribution map.

Figure 4 shows the microstructural response in the upper region (zone 1 in Figure 3) of friction stir additive manufactured AA5A06; Figure 4a depicts the microstructure. According to Figure 4a, the upper structure of the AA5A06 FSAM is composed of fine grains. Notably, there is a band of even finer grains in the middle region of Figure 4a, which corresponds to the interface between the layers. During the FSAM process, the metal between the layers undergoes more intense deformation compared to the metal within the layers, resulting in finer grains. Meanwhile, the metal within the layers has a relatively longer heating time, and the grains observed after stirring are coarser compared to the metal between the layers.

Figure 4b presents the misorientation distribution statistics of the microstructure shown in Figure 4a. The misorientation distribution is primarily concentrated at high-angle grain boundaries, indicating that the fine-grained microstructure in this region is mainly composed of recrystallized grains. Of course, there is also a certain proportion of low-angle grain boundaries, suggesting that some of the fine grains at this region are deformed grains. Figure 4c further statistically analyzes the grain size distribution in the upper region. According to the statistical results, the average grain size in this area is 6.2 μm.

**Figure 5:**
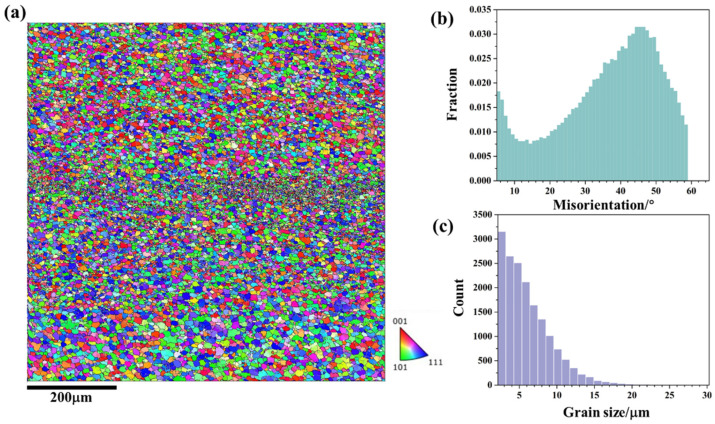
Microstructural response at middle region (zone 2 in Figure 3) of friction stir additive manufactured AA5A06: (**a**) IPF map, (**b**) misorientation distribution map, and (**c**) grain size distribution map.

**Figure 6:**
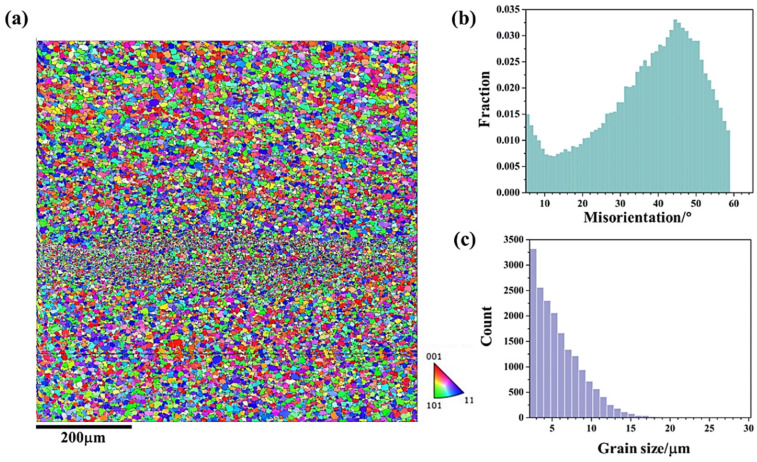
Microstructural response at lower region (zone 3 in Figure 3) of friction stir additive manufactured AA5A06: (**a**) IPF map, (**b**) misorientation distribution map, and (**c**) grain size distribution map.

Figure 5 and Figure 6 show the microstructural responses at the middle region (zone 2 in Figure 3) and lower region (zone 3 in Figure 3) of friction stir additive manufactured AA5A06, respectively. Similarly, the microstructure of the middle region and the lower region is also composed of fine recrystallized grains and deformed grains, and the microstructure of the interface between the layer is also a band of even finer grains. During the FSAM process, the metal between the layers undergoes more intense deformation compared to the metal within the layers, resulting in finer grains. Meanwhile, the metal within the layers has a relatively longer heating time, and after stirring, the grains are coarser compared to those in the metal between the layers. According to the statistical results shown in Figure 5c and Figure 6c, the average grain size of the middle region and lower region of friction stir additive manufactured AA5A06 is 6.1 μm and 5.8 μm, respectively. Therefore, the microstructure of the AA5A06 friction-stir additive structure is relatively consistent and homogeneous, with similar microstructural characteristics and grain sizes in the upper, middle, and lower regions. As a result, it can be expected that the mechanical properties of the upper, middle, and lower regions of the AA5A06 aluminum alloy friction-stir additive structure should also exhibit consistency.

**Figure 7:**
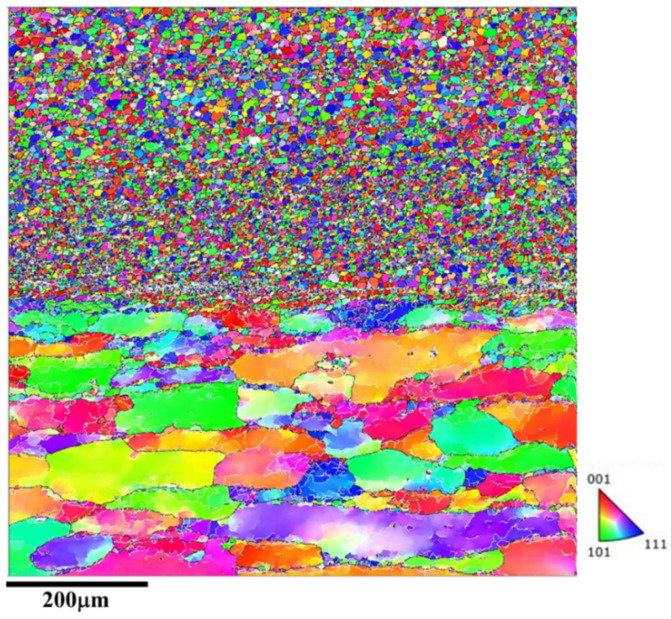
Microstructural response at the interface between the additive structure and the substrate (zone 4 in Figure 3).

The microstructure at the interface between the additive structure and the substrate is shown in Figure 7. It can be observed that a fine FSAM microstructure has formed on the coarse microstructure of the substrate, which indicates the FSAM process has minimal impact on the microstructure of the substrate. Therefore, by using the stir tool, effective FSAM can be achieved.

### 3.3. Tensile Properties of Friction Stir Additive Manufactured AA5A06

**Figure 8:**
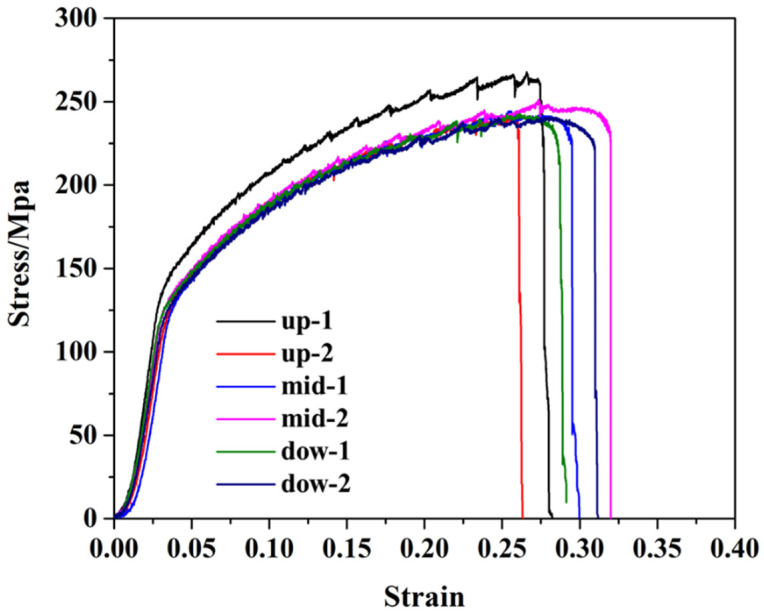
Tensile properties of friction stir additive manufactured AA5A06.

The tensile properties of the AA5A06 friction-stir additive structure in the upper, middle, and lower regions are shown in Figure 8. Two tensile specimens were prepared from each region to obtain reliable performance data. The results show that the performance in the upper, middle, and lower regions of the AA5A06 additive structure is comparable, with tensile strength ranging from 225 MPa to 260 MPa and elongation between 26% and 32%. Based on the above discussion, the microstructure of the AA5A06 friction-stir additive structure in the upper, middle, and lower regions is similar, with an average grain size of ~6 μm. Therefore, the performance is also correspondingly similar.

### 3.4. Morphology of AA5A06 in FSAM with Carbon Nanotubes Added

**Figure 9:**
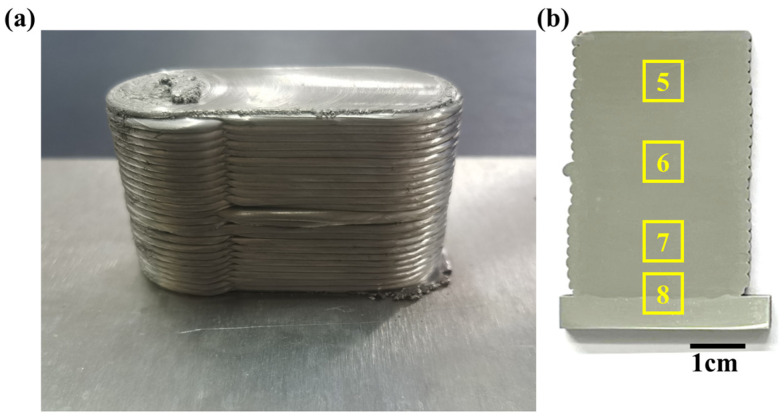
Morphology of AA5A06 in FSAM with carbon nanotubes added: (**a**) appearance, and (**b**) cross-sectional morphology.

The macro morphology of AA5A06 in FSAM with carbon nanotubes added is shown in Figure 9; the appearance is shown in Figure 9a and the morphology of the cross-section is shown in Figure 9b. Compared to the morphology of the AA5A06 friction-stir additive structure shown in Figure 3, the surface of the AA5A06 additive structure with carbon nanotubes added is smoother (Figure 9a). Additionally, as can be seen via the cross-sectional view (Figure 9b), the defects at the edges caused by the friction-stir process have also been significantly improved.

### 3.5. Microstructural Responses of AA5A06 in FSAM with Carbon Nanotubes Added

**Figure 10:**
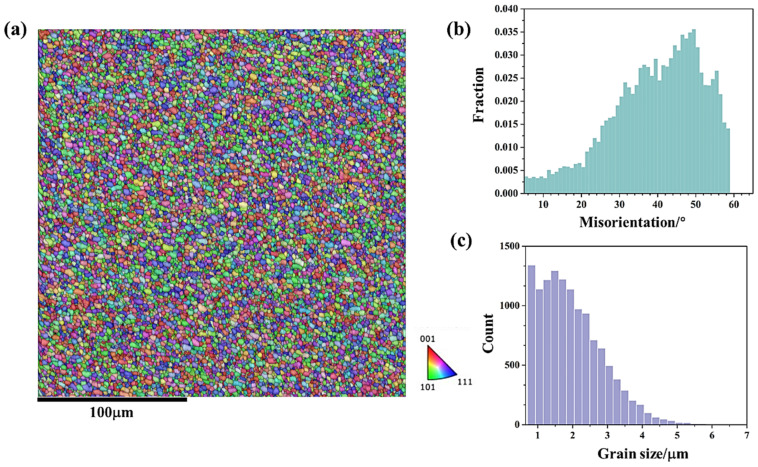
Microstructural response at upper region (zone 5 in Figure 9) of AA5A06 in FSAM with carbon nanotubes added: (**a**) IPF map, (**b**) misorientation distribution map and (**c**) grain size distribution map.

Figure 10 shows the microstructural response at upper region (zone 5 in Figure 9) of AA5A06 in FSAM with carbon nanotubes added, where Figure 10a presents the microstructure. According to Figure 10a, the upper structure is composed of refined grains. Figure 10b presents the misorientation distribution statistics of the microstructure shown in Figure 10a. The misorientation distribution is concentrated at high-angle grain boundaries, indicating that the fine-grained microstructure in this region is mainly composed of recrystallized grains. Figure 10c further statistically analyzes the grain size distribution in the upper region. According to the statistical results, the average grain size in this area is 2.0 μm. As one may have noticed, the band of even finer grains at the interface between layers is hard to find here. This is likely because the carbon nanotubes have sufficiently refined the additive structure (~2 μm), making it difficult to distinguish between the microstructure within the additive layers and that between the layers.

**Figure 11:**
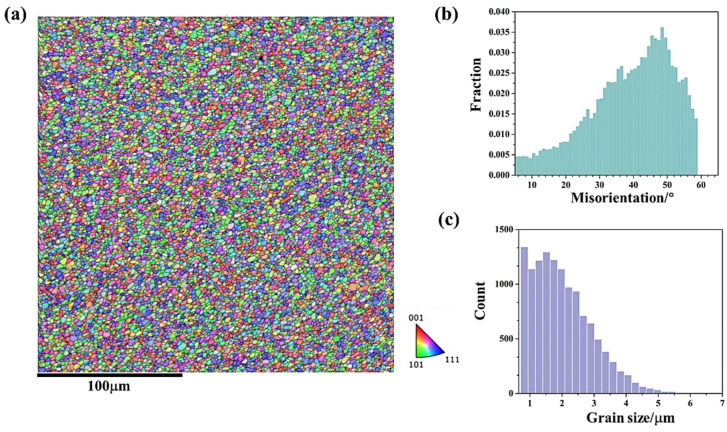
Microstructural response at middle region (zone 6 in Figure 9) of AA5A06 in FSAM with carbon nanotubes added: (**a**) IPF map, (**b**) misorientation distribution map, and (**c**) grain size distribution map.

**Figure 12:**
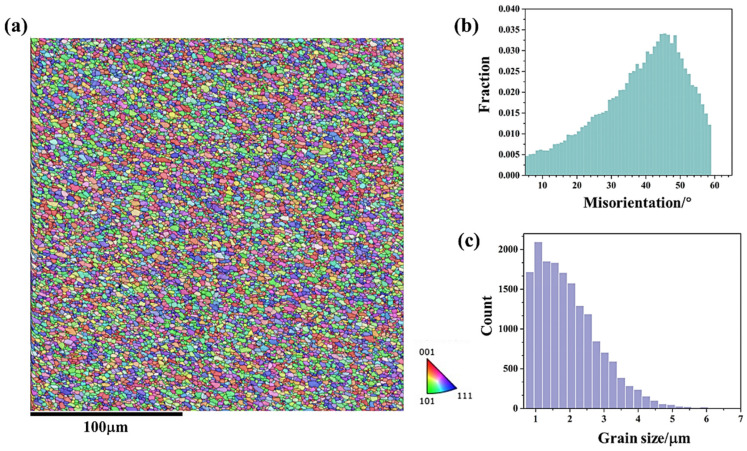
Microstructural response at lower region (zone 7 in Figure 9) of AA5A06 in FSAM with carbon nanotubes added: (**a**) IPF map, (**b**) misorientation distribution map, and (**c**) grain size distribution map.

Figure 11 and Figure 12 show the microstructural responses at the middle region (zone 6 in Figure 3) and lower region (zone 7 in Figure 3) of AA5A06 in FSAM with carbon nanotubes added, respectively. Similarly, the microstructure of the middle region and the lower region is also composed of fine recrystallized grains. According to the statistical results shown in Figure 11c and Figure 12c, the average grain size of the middle region and lower region is 1.9 μm and 2.0 μm, respectively. Compared with the microstructure of the AA5A06 friction-stir additive structure, the microstructure of AA5A06 in FSAM with carbon nanotubes added is more consistent and homogeneous without the band of fine grains at the interface of the layers. Due to the finer grains of the AA5A06 friction-stir additive material with added carbon nanotubes, higher strength can be expected.

**Figure 13:**
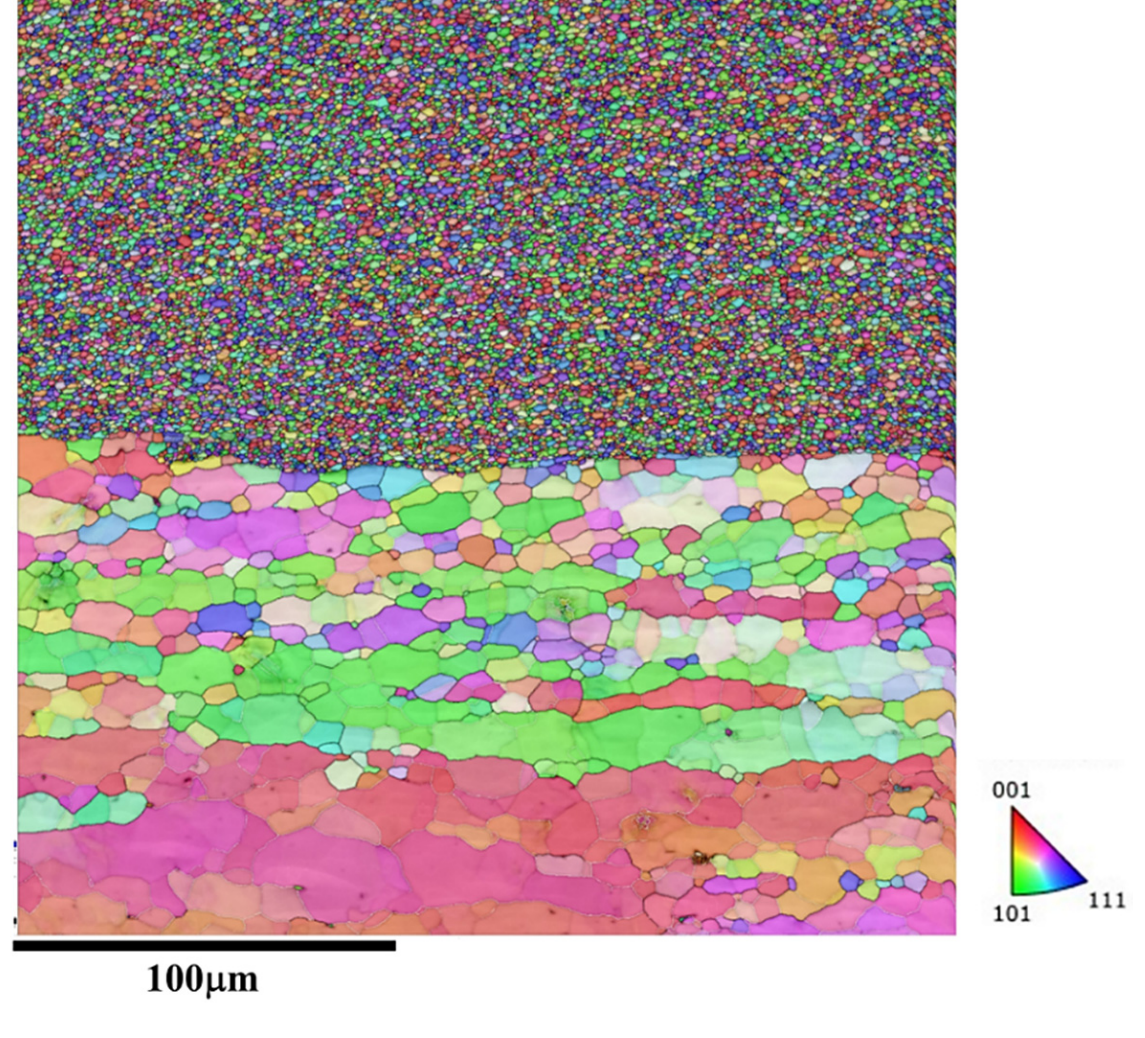
Microstructural response at the interface between the additive structure with carbon nanotubes added and the substrate (zone 8 in Figure 9).

The microstructure at the interface between the additive structure and the substrate is shown in Figure 13. Similarly to the microstructure at the interface of friction stir additive manufactured AA5A06, the fine microstructure of the FSAM has formed on the coarse microstructure of the substrate.

### 3.6. Tensile Properties of AA5A06 in FSAM with Carbon Nanotubes Added

**Figure 14:**
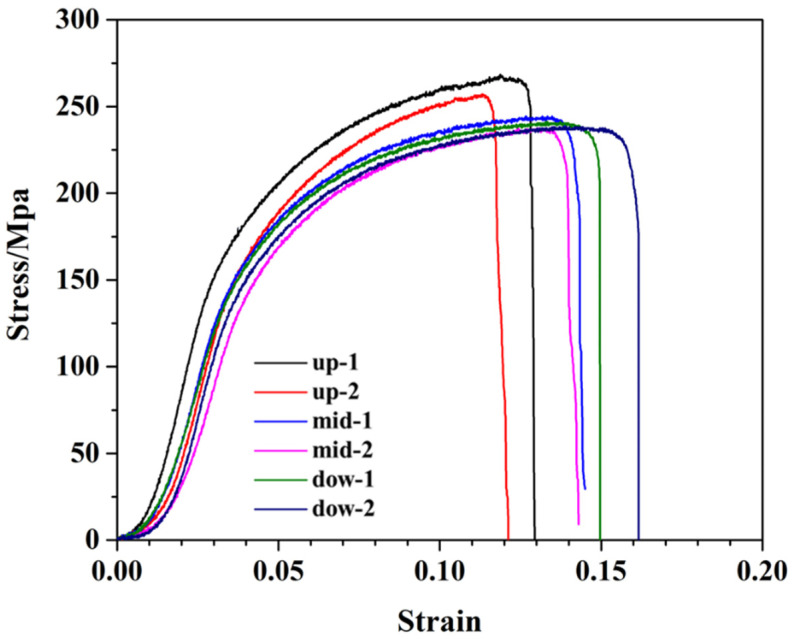
Tensile properties of AA5A06 in FSAM with carbon nanotubes added.

The tensile properties of AA5A06 in FSAM with carbon nanotubes added in the upper, middle, and lower regions are shown in Figure 14. The results show that the performance in the upper, middle, and lower regions of the AA5A06 additive structure with carbon nanotubes added is comparable, with tensile strength ranging from 225 MPa to 270 MPa and elongation between 12% and 16%. Based on the above discussion, although the added carbon nanotubes can further refine the grains of AA5A06 additive material from ~6 μm to ~2 μm, the tensile strength of the structure has not improved, and the elongation has even decreased. This may be because, for sufficiently fine-grained additive structures of AA5A06, the ultimate tensile strength may be limited to around 250 MPa. Adding carbon nanotubes and further refining the grains does not improve the strength.

### 3.7. Comparison Between FSAM and FSAM with Carbon Nanotubes Added

Table 1 compares the microstructure and tensile properties between FSAM and FSAM with carbon nanotubes added. As discussed above, when carbon nanotubes are added during the FSAM process, the average grain size of the aluminum alloy additive decreases from ~6 µm to ~2 µm, but the strength of the additive structure is not improved. This may be because the fully refined grain structure of the additive material has a limited tensile strength of 250 MPa. At the same time, the elongation is reduced because, as the grain size decreases, dislocations are more likely to accumulate at grain boundaries during deformation, leading to damage and a reduction in plasticity. It is worth noting that after the addition of carbon nanotubes, the microstructure of the metal within the layers and the metal between the layers of the additive structure show almost no difference, exhibiting greater uniformity compared to the unmodified material. Therefore, in structures that require long-term service, the deformation of the metal within the layers and the metal between the layers in the aluminum alloy direct friction stir additive structure may be incompatible. The addition of carbon nanotubes may increase the stability of the additive structure during service. Thus, aluminum alloy direct friction stir additive structures, with high elongation, are suitable for applications that require significant deformation. However, due to the uneven structure, mechanical properties may vary in different regions, affecting the overall reliability of the material. This makes it suitable for applications that require high elongation, such as automotive and aerospace components that require substantial deformation capacity, but it may not be suitable for high-fatigue environments. Aluminum alloy friction stir additive structures with added carbon nanotubes, which are more uniform and stronger, are ideal for applications requiring high strength and high fatigue resistance, such as aerospace, automotive components, and military equipment. They are also suitable for applications requiring high material strength with relatively little deformation, such as reinforcing structures and components that endure long-term loads. Future research can further optimize the amount of carbon nanotube addition and its dispersion uniformity to increase strength while maintaining the material’s ductility. The exploration of other nanoscale reinforcing phases, such as graphene, carbon fibers, and their composite effects with aluminum alloys, can further enhance the balance between strength and ductility.

### 3.8. Comparison Between FSAM and Solidification-Based AM

Table 2 summarizes and compares the microstructure, properties, defects, and other characteristics of aluminum alloy FSAM and solidification-based AM (i.e., L-PBF, WAAM) to highlight the advantages, disadvantages, and potential applications of FSAM. First, compared to solidification-based AM processes, FSAM’s advantages lie in its dense microstructure, which can avoid solidification-related defects (i.e., gas porosity, voids, hot cracking), and its ability to achieve a more uniform, finer-grained structure, maintaining high tensile strength. Therefore, aluminum alloy structures formed by FSAM are expected to have greater long-term stability in terms of properties such as fatigue and creep resistance. As a result, FSAM-formed aluminum alloys are more likely to be used in aerospace applications, while solidification-based additive manufacturing of aluminum alloys is more commonly used in automotive and marine applications, among other fields.

## 4. Conclusions

This study implemented a consumable-tool-based 5A06 FSAM process. Our research involved incorporating carbon nanotubes during the FSAM process and investigating the subsequent impact on grain refinement and the performance of the additive structure. Additionally, the uniformity of the microstructure and properties in the upper, middle, and lower regions of the 5A06 FSAM process was analyzed. The following conclusions were drawn:

(1) Based on the consumption-based stirring tool method of FSAM, a well-formed additive structure can be obtained. The structure is composed of multiple layers of stirred metal. At the same time, the microstructure and performance consistency of the additive structure across the upper, middle, and lower regions are relatively good.
(2) The microstructure of the additive structure of AA5A06 consists of refined recrystallized grains and deformed grains within each layer, while the interface between layers is composed of a finer grain band, with an average grain size of 6 µm. After the addition of carbon nanotubes, the grains in the additive structure of AA5A06 are further refined, and the structure is predominantly made up of recrystallized grains, with an average grain size of 2 µm.
(3) The tensile strength of the additive structure of 5A06 aluminum alloy ranges from 225 MPa to 260 MPa, with an elongation of 26% to 32%. After the addition of carbon nanotubes, although the grain size was refined, there was no improvement in tensile strength, and the elongation was reduced. The tensile strength now ranges from 225 MPa to 270 MPa, with elongation between 12% and 16%. This may be because the fully refined grain structure of the additive material has a limited tensile strength of 250 MPa.

## Figures and Tables

**Table 1:** Comparison between FSAM and FSAM with carbon nanotubes added.

	FSAM	FSAM with Carbon Nanotubes Added
**Comparison of microstructure**	** 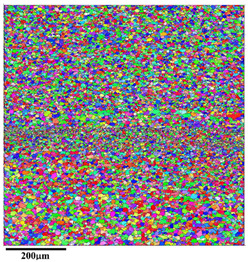 **	** 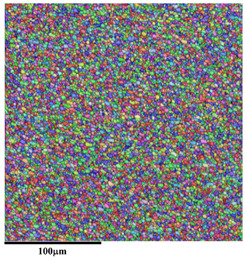 **
**Comparison of properties**	** 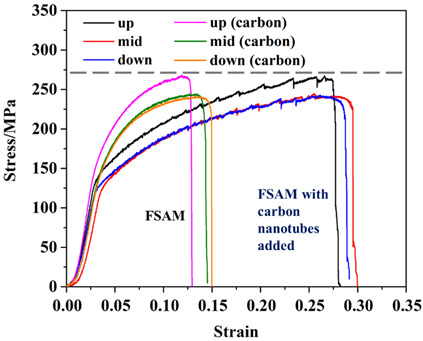 **	

**Table 2:** Comparison between FSAM and solidification-based AM.

Build Process	FSAM (This Study)	FSAM with Carbon Nanotubes (This Study)	L-PBF [28]	WAAM [29,30,31]
Microstructure	Fine grains within the layers and even finer grains between the layers	Refined grains	Epitaxial columnar grains	Coarse equiaxed grains
Grain size	~6 μm	~2 μm	10~100 μm	10~100 μm
Defects type	Dense	Dense	Gas porosity, voids, hot cracking	Gas porosity, voids, hot cracking
Tensile Strength/MPa	Around ~250 MPa	Around ~250 MPa	100~350 MPa	250~300 MPa
Elongation/%	26~32%	12~16%	10~30%	20~30%
Cost	Moderate	High	High	Lower
Application prospects	Aerospace	Aerospace	Automotive trim, boathulls, architecturalcomponents	Automotive trim, boathulls, architecturalcomponents
**Microstructure image**	** 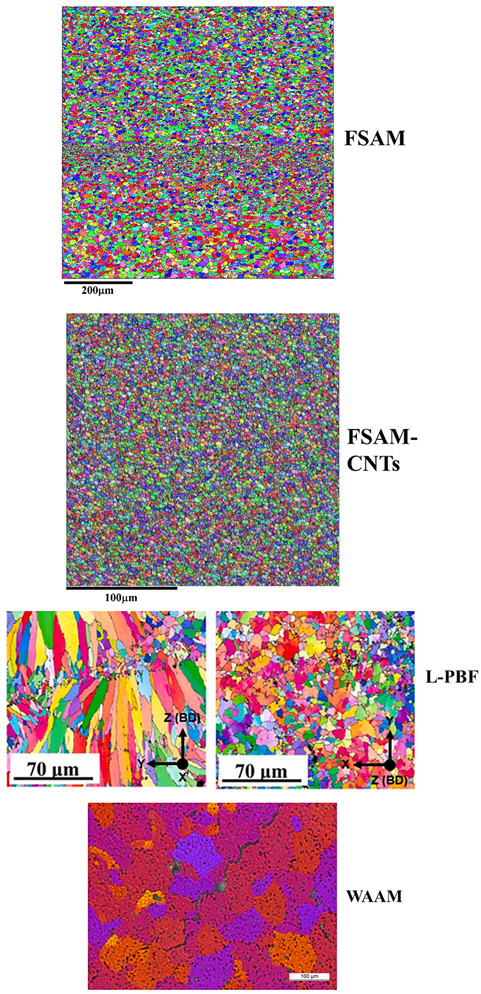 **			

## Data Availability

The original contributions presented in this study are included in the article. Further inquiries can be directed to the corresponding author.
